# Cell surface immune receptors: the guardians of the plant’s extracellular spaces

**DOI:** 10.1016/j.pbi.2019.02.005

**Published:** 2019-08

**Authors:** Kostya Kanyuka, Jason J Rudd

**Affiliations:** Biointeractions and Crop Protection, Rothamsted Research, Harpenden, United Kingdom

## Abstract

Since the original ‘Zigzag model’, several iterations have been proposed to reconcile both the Pattern Triggered Immunity (PTI) and the Effector Triggered Immunity (ETI) branches of the plant immune system. The recent cloning of new disease resistance genes, functioning in gene-for-gene interactions, which structurally resemble cell surface broad spectrum Pattern Recognition Receptors, have further blurred the distinctions between PTI and ETI in plant immunity. In an attempt to simplify further the existing conceptual models, we, herein, propose a scheme based on the spatial localization of the key proteins (receptors) which function to induce plant immune responses. We believe this ‘Spatial Invasion model’ will prove useful for understanding how immune receptors interact with different pathogen types which peripherally or totally invade plant cells, colonize solely extracellularly or switch locations during a successful infection.

## Highlights

• Current plant immunity models lack a simple spatial dimension.
• A new ‘Spatial Invasion model’ of plant immunity is proposed.
• Wall-associated receptor kinases are important new players in immunity in monocots.

**Current Opinion in Plant Biology** 2019, **50**:1–8

This review comes from a themed issue on **Biotic interactions**

Edited by **Rebecca S Bart** and **Ken Shirasu**

For a complete overview see the Issue and the Editorial

Available online 9th March 2019

**https://doi.org/10.1016/j.pbi.2019.02.005**

1369-5266/© 2019 The Authors. Published by Elsevier Ltd. This is an open access article under the CC BY license (http://creativecommons.org/licenses/by/4.0/).

## Introduction

The principal concepts of plant immunity and the general model proposed by Jones and Dangl known as the ‘Zigzag model’ [1^••^], incorporating these concepts, have been formulated in the early to mid-2000’s. These were largely based on the findings from studies of plant interactions with a relatively small number of mainly biotrophic pathogens, that is, those that do not kill their respective hosts and are equipped to utilize nutrients provided by the living plant cell. It is becoming increasingly difficult to fit the new findings from studies of diverse pathosystems, particularly those that involve pathogens that thrive outside of the host cells, into the original model of plant immunity. Here, we highlight the limitations of this model and inconsistences in the terminologies used to describe specific components or features of the plant immune system existing in the current literature. We will then touch upon the recently proposed alternative model of plant immunity termed the ‘Invasion model’ [2^••^] proposed just over three years ago, which in our view is more inclusive and applicable to a wider range of plant–microbe interactions. However, we suggest a further simplification, refocusing only on plant interactions with pathogens, and further refinement by introducing a spatial categorization of plant immune receptors. Finally, we will draw a special attention to one particular structural class of cell surface located immune receptors, namely Wall-Associated Kinase-like (WAK) proteins [3], whose members were previously described as pectin receptors. With several new gene members cloned in recent years from different cereal crops directly implicated in control of broad-spectrum or isolate/race-specific resistance to various plant pathogens, WAKs are emerging as new important players in plant immunity.

## Key concepts of plant immunity and the original ‘Zigzag model’

Two branches of the plant immune system are recognized: PTI (Pattern-Triggered Immunity) and ETI (Effector-Triggered Immunity) [1^••^]. PTI is considered to be based upon conserved plasma membrane-associated extracellular Pattern-Recognition Receptors (PRRs) [4,5^•^], such as Receptor-Like Kinases (RLKs) and Receptor-Like Proteins (RLPs; similar to RLKs but missing a cytoplasmic kinase domain), detecting highly conserved microbial features (a.k.a. Pathogen-Associated Molecular Patterns, PAMPs) such as bacterial cell wall-derived peptidoglycans or flagella fragments in the host apoplast. PTI is often (but not always) achieved without the death of the affected plant cells. Successful biotrophic pathogens deliver a battery of secreted proteins contributing to virulence, knowns as effectors, inside the host cell where some act to suppress PTI and others reprogram host cell metabolism and physiology to aid host colonization [6,7]. Individual genotypes within the same host species may possess highly variable and often dispensable intracellular receptors known as disease resistance (R) proteins that can sense specific effectors or monitor their activities, resulting in the activation of ETI. This is often but not always associated with programmed cell death of the affected cell, otherwise known as a hypersensitive response (HR) [8,9]. The large majority of R proteins studied to date belong to the NB-LRR (Nucleotide Binding Site-Leucine Rich Repeat domain) class [1^••^,^10^]. When a particular R protein becomes widespread among host genotypes (e.g. through breeding and/or agriculture), this puts the pathogen under pressure to accumulate mutations in the corresponding effector protein or to lose the effector entirely, thus avoiding ETI [11]. The pathogen may also evolve new effector(s) able either to suppress ETI triggered by another effector or decoy effector(s) that are sensed by the same R protein without activation of ETI [12]. In turn, plants can evolve either new R proteins recognizing new effector variants or decoy proteins mimicking the true effector targets whose status can be sensed by the existing R proteins [12]. This evolutionary arms race between plants and their pathogens appears to continue indefinitely [1^••^]. PTI is considered to confer resistance to a broad spectrum of pathogens or lineages of pathogens and, as previously mentioned, it is often thought of as a weak defense response, infrequently associated with HR. By contrast, ETI is thought to confer a narrower isolate-specific or strain-specific resistance, also known as gene-for-gene resistance [13], which is rapid, strong/intense, and often culminates in HR.

## Limitations of the original model

The original model omits consideration of evidence that host defenses could also be triggered by endogenous plant-derived molecules known as Damage-Associated Molecular Patterns (DAMPs) [14,15], including cutin monomers or cell-wall derived oligogalacturonides (OGs) released into the apoplast due to the action of various pathogen-secreted cell wall degrading and other lytic enzymes, and secreted endogenous peptides produced in response to pathogen attack. To date, less than a handful of DAMP receptor genes have been isolated and characterized, but all appear to encode RLKs [16, 17, 18] or, in one case, a WAK protein [19]. These PRRs could easily be incorporated into the original model, especially since each protein seems to activate immune responses similar to those triggered by PAMPs. However, other new findings from studies of diverse pathosystems are becoming increasingly difficult to fit into the original model of plant immunity. Moreover, at least some of the original concepts and definitions are being challenged by the growing experimental data suggesting that there may be no clear distinction between PTI and ETI or PAMPs and effectors. This view was first voiced in 2011 by Thomma *et al.* [20], who provided a number of compelling examples of typical effectors that show, similar to PAMPs, a high level of sequence conservation within and even between the different pathogen species and PAMPs that exhibit, similar to effectors, at least some sequence diversity and a narrow distribution across the pathogen species. Other scientists have pointed out that PTI and ETI both can be robust or weak, depending on the specific interaction, and that activation of HR can be separated from activation of pathogen resistance [21, 22, 23]. Also, PTI triggered by certain PAMPs can result in HR [20]. Further, there are examples of intracellular R proteins, such as barley RPG1 (kinase with tandem kinase domains) and wheat WKS1 (kinase containing a START lipid binding domain), which confer resistance to the stem and stripe rust fungi, respectively, and show remarkable sequence conservation akin to that of PRRs [24, 25, 26].

Not all pathogen effectors, even those of biotrophic pathogens, are delivered or translocated inside the host cell cytoplasm and some could be recognized in the apoplast by extracellular receptors structurally similar to PRRs. In addition, a number of fungal pathogens that cause serious diseases of crop plants colonize extracellular spaces and do not form specialized feeding structures or penetrate host cells either during entire life cycle or at least during prolonged initial phases of infection, and, therefore, these probably produce largely apoplast located effectors [27,28]. Indeed, effectors of these pathogens have been isolated from the apoplastic fluid or xylem sap of infected tissue and recognition of some of these effectors in the apoplast rather than cytoplasm have been demonstrated. The most well-known of these, and also featured in the original Zigzag model, are Avr2, Avr4, Avr5, and Avr9 of *Cladosporium fulvum* (recently renamed to *Passalora fulva*), a fungal pathogen that causes tomato leaf mold [29]. Perhaps unsurprisingly, these are recognized by the PRR-like RLPs Cf-2, Cf-4, Cf-5, and Cf-9 (Table 1), respectively, rather than by the NB-LRR class of R proteins. Nevertheless, plant defense induced by Cf proteins is often referred to as ETI [1^••^]. This is somewhat confusing. Alternatively, Cf proteins as well as other immune receptors that recognize extracellular located pathogen effectors are sometimes classified as PRRs [5^•^], which we think is equally confusing because each has a very narrow recognition specificity. Also, because transfer of some *bona fide* PRRs from one plant family to another may result in partial or even complete resistance [30] these cell surface receptors sometimes are referred to as R proteins [31^•^]. Finally, in an attempt to distinguish resistance conferred by the cell-surface immune receptors recognizing effectors of apoplastic pathogens from ETI, a term ‘ETD’ (‘effector-triggered defense’) [27] has been proposed. This, we think, is also fairly confusing because ‘immunity’ and ‘defense’ are considered to be synonymous as ‘immunity’ is defined as a ‘host defense system’. Plus, in our opinion, host defenses triggered by the apoplastic effectors shouldn’t be considered distinct from those induced by the typical PAMPs as both are orchestrated by the structurally similar cell surface receptors and, therefore, likely involve activation of similar signaling pathways.

**Table 1:** Cloned genes for resistance to extracellular fungal pathogens

Resistance gene		Plant	Pathogen	Invasion molecule	Reference
Name	Class				
*I*	LRR-RLP	*Solanum pimpinellifolium*	*Fusarium oxysporum* f. sp. *lycopersici*	Avr1 (Six4)	[51]
*I-2*	NB-LRR	*S. pimpinellifolium*	*F. oxysporum* f. sp. *lycopersici*	Avr2 (Six3)	[52]
*I-3*	S-RLK^a^	*Solanum pennellii*	*F. oxysporum* f. sp. *lycopersici*	Avr3 (Six1)	[53]
*I-7*	LRR-RLP	*S. pennellii*	*F. oxysporum* f. sp. *lycopersici*	unknown	[54]
*Fom-1*	NB-LRR	*Cucumis melo*	*F. oxysporum* f. sp. *melonis*	unknown	[55]
*Fom-2*	NB-LRR	*C. melo*	*F. oxysporum* f. sp. *melonis*	AVRFOM2	[56]
*RFO1*	WAK	*Arabidopsis thaliana*	*F. oxysporum* f.sp. *matthioli* *F. oxysporum* f.sp. *raphani*	unknown	[38]
*RFO2*	LRR-RLP	*A. thaliana*	*F. oxysporum* f.sp. *matthioli*	unknown	[57]
*RFO3*	S-RLK	*A. thaliana*	*F. oxysporum* f.sp. *matthioli*	unknown	[58]
*Ve1*	LRR-RLP	*Solanum lycopersicum*	*Verticillium dahlia* *Verticillium albo-atrum* *F. oxysporum* f. sp. *lycopersici*	Ave1	[37]
*Rvi6 (HcrVf2)*	LRR-RLP	*Malus floribunda*	*Venturia inaequalis*	unknown	[59]
*Rvi15 (Vr2-C)*	NB-LRR	*M. floribunda*	*V. inaequalis*	unknown	[60]
*LepR3 (Rlm2)*	LRR-RLP	*Brassica napus*	*Leptosphaeria maculans*	AvrLm1 (AvrLm2)	[61,62]
*Stb6*	WAK	*Triticum aestivum*	*Zymoseptoria tritici*	AvrStb6	[36^•^]
*Cf-2*	LRR-RLP	*S. pimpinellifolium*	*Passalora fulva*	Avr2	[32]
*Cf-4*	LRR-RLP	*Solanum hirsutum*	*P. fulva*	Avr4	[35]
*Cf-5*	LRR-RLP	*S. lycopersicum*	*P. fulva*	Avr5	[33]
*Cf-9*	LRR-RLP	*S. pimpinellifolium*	*P. fulva*	Avr9	[34]
*Hcr9-4E*	LRR-RLP	*Solanum hirsutum*	*P. fulva*	Avr4E	[35,63]

a S-domain receptor-like kinase; S-domain is homologous to the self-incompatibility-locus glycoproteins of *Brassica oleracea.*

## The new ‘Invasion model’ and its simplification and further refinement

**Figure 1:**
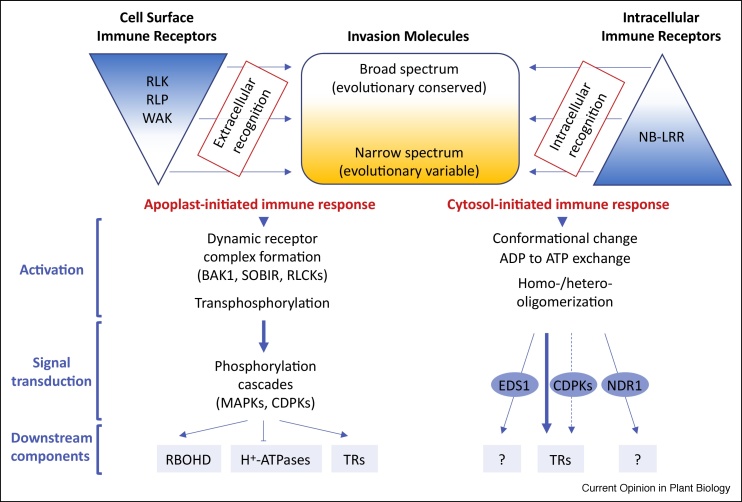
Proposed ‘Spatial Invasion model’ of plant immunity.

To address the limitations and inconsistences stated above, an alternative model of plant immunity, termed the ‘Invasion model’, in which host receptors (termed ‘Invasion Pattern Receptors’) detect either microbe-encoded or host-derived ligands that indicate invasion (termed ‘Invasion Patterns’ or ‘IPs’), has been proposed [2^••^]. According to this model, any molecule could serve as an IP and potentially be detected by an immune receptor. This model also proposes that all classes of immune receptors could induce either a weak or strong immune response, be phylogenetically conserved or variable, confer immunity to a narrow or broad range of invaders, and engage either specific or more common signaling pathways and components. In addition, the Invasion model considers that some IP-triggered responses do not necessarily result in immunity. This more general model aimed to describe all interactions involving plants and their pathogens/pests as well as endophytic and mutualistic organisms, which is commendable but, in our view, makes the Invasion model somewhat too complex. We endorse these views and some of the terms/definitions but suggest (i) limiting this model to cover interactions of plants only with their adapted pathogens, and (ii) introducing a spatial dimension to the model reflecting the fact that the immune receptor-IP recognition could take place either outside (apoplast) or inside the host cell. Importantly, we propose to recognize two spatially separated immune receptor types, Cell Surface Immune Receptors (CSIRs) and Intracellular Immune Receptors (IIRs), which trigger mechanistically distinct defenses upon direct or indirect recognition of apoplastic or cytoplasmic ‘Invasion molecules’ or ‘IMs’ (see Glossary, and Figure 1). This revised and simplified model of plant immunity, which we term ‘Spatial Invasion model’, is less inclusive than the Invasion model but extends more than the Zigzag model to cover a wide range of plant-microbe interactions including those that involve pathogens that thrive outside of host cells. These include many *Dothideomycete* fungi that colonize apoplastic spaces and grow in close contact with the leaf mesophyll cells but never or rarely penetrate, such as *Zymoseptoria tritici*, *Leptospaeria maculans*, *Mycosphaerella fijiensis*, and *P. fulva*—causal agents of important foliar diseases of wheat, oilseed rape, banana and tomato, respectively. These also include species such as *Rhynchosporium commune, Pyrenopeziza brassicae* and *Venturia inaequalis* that grow subcuticularly in close contact with the leaf epidermal cells and induce serious diseases in barley, oilseed rape, and apple, respectively. In addition, several fungal species including *Fusarium oxysporum* and *Verticillium dahlia* colonize the plant vascular system, namely xylem vessels, and cause economically important wilt disease in tomato and several other crops. Evidence suggests that these extracellular pathogens are likely to be recognized primarily by CSIRs in the apoplast (Table 1) resulting in either race-specific [32, 33, 34, 35,36^•^] or broad spectrum [37,38] resistance.

## WAK’s – a diverse family of CSIRs of particular importance for monocot plants

The WAK class of CSIRs is specific to the Plant Kingdom and deserves special mention. This is particularly because individual WAKs have been shown to recognize diverse IMs of either plant or microbial origin (i.e. those originally defined as DAMPs, PAMPs, and effectors) and orchestrate either pathogen nonspecific/broad-spectrum immunity or be engaged in gene-for-gene interactions. One of the first and the most well characterized WAKs is the Arabidopsis WAK1, which has been shown to bind plant cell wall pectin as well as pectin break-down products, oligogalacturonides (OGs), generated during pathogen attack, and to activate plant immune responses [19,39,40]. Another WAK protein in Arabidopsis, RFO1 [38], confers resistance to several *formae speciales* of *F. oxysporum* suggesting this CSIR may be recognizing a conserved IM. In contrast, the recently cloned wheat gene *Stb6*, which encodes a WAK protein, confers resistance only to those isolates of *Z. tritici* that express a particular isoform of a matching small secreted protein AvrStb6 [36^•^,^41^,^42^]. Thus, members of the WAK family have now been shown to confer broad spectrum as well as pathogen race-specific resistances. The Arabidopsis genome contains 5 genes annotated as WAKs and 22 additional genes annotated as WAK-like genes [43]. The WAK gene family shows dramatic expansion in monocots and comprises ∼130 members in rice [44,45] and over 600 members in wheat [46]. This suggests that cell wall to cytoplasm communication may play an important role in the biology of monocots, including in pathogen defense. Whilst further research is clearly needed, we propose that that these CSIRs monitor changes in the plant cell wall, including those caused by the activity of pathogens, and transmit signals to the cytoplasm/nucleus to initiate various defense and/or growth and development processes such as cell expansion, strengthening of cell wall, for example, through lignification and other types of polymer depositions. Necrotrophic pathogens in particular, secrete numerous Cell-Wall Degrading Enzymes (CWDEs) such as pectinases, cellulases, xylanases, and cutinases and some WAKs appear to have evolved ability to recognize cell-wall derived molecules (e.g. OGs) released by CWDEs, and to function in plant immunity. Several major genes conferring resistance to fungal or bacterial pathogens that encode WAKs have been recently cloned from wheat (*Stb6*), maize (*Htn1* and *qHSR1*) and rice (*Xa4*) [36^•^,47^•^,48^••^,49^••^]. IMs recognized by Htn1 and qHSR1 are not known, but these are likely to be quite conserved pathogen produced molecules, as these immune receptors confer a quantitative type of disease resistance. Rice Xa4, similarly to wheat Stb6, controls a race-specific resistance and, therefore, probably recognizes a less conserved bacterial effector protein. Alternatively, because Xa4 reduces plant height by strengthening the plant cell wall even in the absence of the pathogen [49^••^], the IM recognized by Xa4 may be of a plant origin. Interestingly, one of the other recently cloned wheat WAK genes, *Snn1*, has been shown to mediate susceptibility to the strains of a necrotrophic fungal pathogen *Parastagonospora nodorum* that produce an apoplastic necrosis-inducing effector SnTox1, and a direct interaction between wheat Snn1 and fungal SnTox1 has been demonstrated [50^••^]. These data, therefore, show that WAK-mediated defense pathways could be targeted/hijacked by necrotrophic pathogens to promote disease. Overall, WAKs are emerging as important new players in cereal disease resistance.

## Conclusions

The immune receptor repertoire of plants is complex and rapidly increasing in both numbers and structural forms. These data permit putative immune receptors to be categorized into those which are more likely to be involved in providing resistance to different pathogen types (or invasion strategies). Previously this was based predominantly on the nutritional lifestyle of the pathogen, but as a further refinement a spatial element can now be included. We propose that broad spectrum cell surface immune receptors contribute to immunity in most, if not all, cases. However, the type of immune receptor which might be engaged in more specific resistances will depend on several features, one of which is the spatial localization of the infection process. Put simply, for pathogens that physically invade plant cells and/or are known to deliver effectors into them, the most likely effective resistance gene type will most frequently involve intracellular NB-LRRs. Conversely for non-cell penetrating apoplastic pathogens and/or where there is no current evidence for transfer or translocation of effectors into plant cells, the specific resistance gene types will be RLKs, RLPs, or WAKs, potentially recruited to these specific functions from the larger original pool of cell surface receptor proteins including those which confer broad spectrum immunity. Further research and resistance gene isolation for more spatially distinct plant–pathogen interactions is required to either substantiate or refute this model.

This new model recognizes two distinct but concurrently operating immune responses that are initiated in the two different compartments – apoplast and cytosol, and mediated by different classes of immune receptors – CSIRs (RLKs, RLPs, or WAKs) and IIRs (mostly NB-LRRs), following recognition of apoplast-located or cytosol-located Invasion molecules (IMs). IMs could be either broadly conserved within or even across species or higher taxa or restricted to specific species or even to individual lineages (e.g. ecotypes, cultivars, isolates, and races) within the species. CSIRs and NB-LRRs are more likely to detect evolutionary conserved and more variable IMs as indicated by the large upside down and the regular triangle, respectively. Importantly, extrapolating from the data available for some well-characterized CSIRs (e.g. FLS2, Cf-proteins) [14,64^•^] and NB-LRR proteins (e.g. MLA10, RPS4) [65,66^•^,67^•^,^68^], we propose that these activate defense signaling through entirely different mechanisms and also engage separate signaling components. The former initiate signaling through a series of plasma membrane-localized phosphorylation/dephosphorylation events and likely engage co-receptor RLKs, such as BAK1 (BRI1-Associated Receptor Kinase) and SOBIR1 (Suppressor of BIR1-1) [69,70] or other types of cell surface receptors, as well as Receptor-Like Cytoplasmic Kinases (RLCKs). The signal is then internalized through the activation of RLCKs, Mitogen-Activated Protein Kinases (MAPKs) cascades, and Ca^2+^-Dependent Protein Kinases (CDPKs), which leads to activation of the NADPH oxidase Respiratory Burst Oxidase Homologue Protein D (RBOHD) responsible for the production of reactive oxygen species and inactivation of the plasma membrane residing H^+^-ATPases resulting in extracellular alkalinization, as well as ultimate stimulation of Transcription Regulators (TRs) that regulate expression of numerous defense genes. By contrast, the precise mechanisms of activation and the signaling pathways leading to defense activation for many known NB-LRRs remain only partially understood. It appears that in the absence of pathogens, NB-LRRs are held in an inactive state, which is facilitated through the intramolecular interactions between their NB (bound to ADP) and LRR domains. Some NB-LRRs are activated following direct binding to the corresponding IMs, whereas activation of others is triggered following interaction with host proteins modified through the action of pathogen produced IMs. In each case, these protein-protein interactions induce a conformational change associated with the ADP to ATP exchange, which frees its N-terminal (coiled-coil or Toll-like/IL-1 receptor) domain promoting an NB-LRR homodimerization and/or heterodimerization or formation of more complex interaction networks with other (‘helper’) NB-LRRs [71^••^,^72^] and initiation of downstream signaling. Exactly how the activated NB-LRRs induce defense signaling pathways is poorly understood. Several characterized NB-LRRs seem to be able to shuttle between the cytoplasm and the nucleus where they activate transcription of defense genes through direct interaction with TRs. Many other NB-LRRs do not seem to reside in or be able to translocate to the nucleus, and instead associate with the plasma membrane or other endomembrane compartments such as the vacuole, Golgi or late endosomes. How these NB-LRRs activate defense responses remains unclear; however, considering the available data for the plasma membrane tethered NB-LRRs (such as *Arabidopsis thaliana* RPM1 and RPS2) this may involve influx of Ca^2+^ and various CDPKs that perceive Ca^2+^ signals and probably translate these into phosphorylation/activation of TRs [65]. Moreover, several NB-LRRs containing an N-terminal Toll-like/IL-1 receptor domain appear to signal through Enhanced Disease Susceptibility 1 (EDS1) [23], whereas some NB-LRRs containing a coiled-coil domain in their N-terminus are thought to engage another signaling protein Non Race-Specific Disease Resistance 1 (NDR1) [73].

## References and recommended reading

Papers of particular interest, published within the period of review, have been highlighted as:

• of special interest
•• of outstanding interest

## Glossary

**Terms used in the proposed ‘Spatial Invasion model’ of plant immunity**
Invasion molecules (IMs): *Sensu stricto* PAMPs, effectors (both apoplastic and cytosolic), and DAMPs, as well as any other pathogen-encoded or plant-encoded evolutionary conserved or variable molecules that signal invasion and trigger immune responses.
Immune receptors: Plant proteins that perceive IMs and orchestrate immune responses, including *sensu stricto* PRRs as well as R proteins.
Cell surface immune receptors (CSIRs): Membrane-associated plant proteins containing domains extending into the extracellular space (such as RLKs, RLPs, and WAKs) that perceive apoplastic IMs.
Intracellular immune receptors (IIRs): Plant proteins located inside the cell (mostly NB-LRRs) that perceive cytosolic IMs.
